# 

**Published:** 1898-09

**Authors:** 


### CONTENTS.

ORIGINAL COMMUNICATIONS. PAGE

Secondary Operation for Harelip. By Dr. Thomas Fillebrown, Boston . . . . 565
Treatment of Cervical Borders. By Safford G. Perry, D.D.S., New York . . 567
Some Properties of Vulcanite Rubber. By S. E. Davenport, D.D.S., M.D.S. . 579
The First Meeting with Patients. By Charles A. Brackett, D.M.D., Newport, R. I. . . . . . . . . . . . . . . . . . . . . . . . . . . . . 582

ABSTRACTS AND TRANSLATIONS.

Why Coagulants diffuse through Dentine. By E. Lawley York, D.D.S., F.R.M.S., England . . . . . . . . . . . . . . . . . . . . . . . . 586

REPORTS OF SOCIETY MEETINGS.

The New York Institute of Stomatology . . . . . . . . . . . . . . 591

Academy of Stomatology . . . . . . . . . . . . . . . . . . . . . . 604

EDITORIAL.

The Tendency to lean on the Past . . . . . . . . . . . . . . . . . 609

BIBLIOGRAPHY . . . . . . . . . . . . . . . . . . . . . . . . . . . 612

OBITUARY.

Dr. William Pepper . . . . . . . . . . . . . . . . . . . . . . . . 618

Dr. Alfred P. Southwick . . . . . . . . . . . . . . . . . . . . . . 621

NOTES AND COMMENTS . . . . . . . . . . . . . . . . . . . . . . . . 622

CURRENT NEWS.

New Jersey State Dental Society . . . . . . . . . . . . . . . . . . 625
Southern Branch of the National Dental Association, Second Annual Meeting, New Orleans, La., February 9, 10, 11, and 13, 1899 . . . . . . . . . . . 627
Southern California Dental Association . . . . . . . . . . . . . . 628
Meeting of the Vermont State Board of Dental Examiners . . . . . . . 628

